# Sex-Specific Cardiac Troponin Thresholds in Transgender Patients With Suspected Acute Coronary Syndrome

**DOI:** 10.1001/jamanetworkopen.2023.37345

**Published:** 2023-10-12

**Authors:** Jiexi Wang, Alison Taur, Aiyu Chen, Yi-lin Wu, Ming-Sum Lee

**Affiliations:** 1 Department of Cardiology, Kaiser Permanente Los Angeles Medical Center, Los Angeles, California; 2 Department of Clinical Science, Kaiser Permanente Bernard J. Tyson School of Medicine, Pasadena, California; 3 Department of Nuclear Medicine, Kaiser Permanente Ontario Medical Center, Ontario, California; 4 Department of Research and Evaluation, Kaiser Permanente Southern California, Pasadena

## Abstract

This cohort study compares the use and outcomes of high-sensitivity cardiac troponin assay vs conventional troponin assay in transgender adults.

## Introduction

Diagnosing acute myocardial infarction (AMI) requires the detection of cardiac troponin above the 99th percentile upper reference limit.^1,2^ The sex-specific 99th percentile cutoff for cisgender males is approximately twice that for cisgender females^3^ and is recommended to improve identification of AMI in cisgender females.^4^ Transgender individuals have gender identities that differ from their sex assigned at birth. Medical transitioning includes gender-affirming hormone therapy.^5^ Exposure to hormone therapy affects levels of cardiac biomarkers.^6^ There is no established cardiac troponin diagnostic threshold for transgender individuals. This study aimed to determine the role of implementing sex-specific troponin thresholds in the diagnosis and management of suspected acute coronary syndrome (ACS) in transgender patients.

## Methods

This population-based cohort study included Kaiser Permanente Southern California (KPSC) members (aged ≥18 years) presenting with suspected ACS between 2007 and 2022. The KPSC Institutional Review Board approved the study and waived the informed consent requirement because the study presented minimal risks. We followed the STROBE reporting guideline.

Transgender patients were identified through review of an internal registry, hormonal treatment history, sex assigned at birth, and medical records. Patients never treated with hormonal therapy were excluded. In 2021, the KPSC health system implemented a high-sensitivity cardiac troponin I (hs-cTnI) assay (Access hsTnI; Beckman) with sex-specific diagnostic thresholds. Prior to 2021, a conventional troponin assay with a single threshold was used. Troponin levels on presentation were identified, and cause of troponin elevation was adjudicated by physicians using the universal definition of myocardial infarction (MI).^2^ Patients were followed up for 30 days for outcomes.

Outcomes before and after the hs-cTnI assay implementation were compared using the χ^2^ test. Two-sided *P* < .05 defined statistical significance. Statistical analyses were conducted with Stata/MP 17.0 (StataCorp LLC).

## Results

Among the 1108 patients included, 661 (59.7%) were transgender women and 447 (40.3%) were transgender men, with a median (IQR) age of 33 (25-47) years. Overall, patients self-identified as Asian (77 [6.9%]), Black (108 [9.8%]), Hispanic (360 [32.5%]), or White (524 [47.3%]). Table 1 provides their baseline characteristics.

**Table 1.** Baseline Characteristics^a^

	Conventional troponin assay, No. (%)		*P* value^b^	hs-cTnI Assay, No. (%)		*P* value^b^
	Transgender women (n = 521)	Transgender men (n = 325)		Transgender women (n = 140)	Transgender men (n = 122)	
Age, median (IQR), y	40 (28-54)	28 (22-37)	<.001	33 (24-48)	31 (25-39)	.007
Race and ethnicity^c^						
Asian	46 (8.8)	11 (3.4)	.003	13 (9.3)	7 (5.7)	<.001
Black	43 (8.3)	46 (14.1)		6 (4.3)	13 (10.7)	
Hispanic	159 (30.5)	104 (32.0)		38 (27.1)	59 (48.4)	
White	256 (49.1)	152 (46.8)		79 (56.4)	37 (30.3)	
Other	17 (3.3)	12 (3.7)		4 (2.9)	6 (4.9)	
Insurance						
Commercial	311 (59.7)	212 (65.2)	.001	84 (60.0)	81 (66.4)	.05
Medicare	66 (12.7)	15 (4.6)		14 (10.0)	3 (2.4)	
Medicaid	60 (11.5)	40 (12.3)		23 (16.4)	16 (13.1)	
Other^d^	84 (16.1)	58 (17.8)		19 (13.6)	22 (18.0)	
Comorbidities						
Hypertension	121 (23.2)	35 (10.8)	<.001	19 (13.6)	14 (11.5)	.61
Diabetes	67 (12.9)	23 (7.1)	.008	9 (6.4)	9 (7.4)	.76
Obesity	112 (21.5)	79 (24.3)	.34	20 (14.3)	28 (22.9)	.07
Heart failure	5 (0.9)	2 (0.6)	.59	1 (0.7)	1 (0.8)	.92
Coronary artery disease	20 (3.8)	0	<.001	3 (2.1)	1 (0.8)	.38
Atrial fibrillation	5 (0.9)	2 (0.6)	.59	2 (1.4)	1 (0.8)	.64
Valvular heart disease	5 (0.9)	1 (0.3)	.27	5 (3.6)	1 (0.8)	.14
Kidney failure	32 (6.1)	6 (1.9)	.003	0	1 (0.8)	.28
COPD or asthma	83 (15.9)	72 (22.2)	.02	30 (21.4)	20 (16.4)	.30
Liver disease	28 (5.4)	6 (1.9)	.01	7 (5.0)	3 (2.5)	.28
Depression	174 (33.4)	115 (35.4)	.55	75 (53.6)	45 (36.9)	.007

Abbreviations: COPD, chronic obstructive pulmonary disease; hs-cTnI, high-sensitivity cardiac troponin I.

^a^ Unpaired, 2-tailed *t* test for continuous variables; χ^2^ test for categorical variables.

^b^ *P* values are given for transgender men vs transgender women.

^c^ Race and ethnicity were self-reported. Other race and ethnicity included undisclosed information, mixed race, and unknown.

^d^ Other insurance included self-insured or unknown.

Two hundred sixty-two patients (23.6%) were evaluated after implementation of the hs-cTnI assay with sex-specific thresholds; all other patients (846 [76.4%]) were evaluated using a conventional troponin assay with a single threshold. With the conventional troponin assay, 24 individuals (2.8%) had abnormal troponin levels (Table 2). With the hs-cTnI assay, abnormal levels were detected in 11 individuals (4.2%) if the threshold used was for their sex assigned at birth and in 10 individuals (3.8%) if the threshold aligned with their affirmed gender identity. The overall concordance for classifying patients into having normal vs abnormal troponin levels was high (98.9%).

**Table 2.** Cardiac Troponin Levels, Abnormal Troponin Levels, and 30-Day Clinical Outcomes

	Transgender women, No. (%)		*P* value^a^	Transgender men, No. (%)		*P* value^a^	All patients, No. (%)		*P* value^a^
	Conventional troponin assay (n = 521)	hs-cTnI Assay (n = 140)		Conventional troponin assay (n = 325)	hs-cTnI Assay (n = 122)		Conventional troponin assay (n = 846)	hs-cTnI Assay (n = 262)	
Abnormal troponin level: >99th percentile^b^									
Using a single threshold	20 (3.8)	NA	NA	4 (1.2)	NA	NA	24 (2.8)	NA	NA
Using the threshold for sex assigned at birth	NA	7 (5.0)	NA	NA	4 (3.3)	NA	NA	11 (4.2)	NA
Using the threshold aligned with affirmed gender identity	NA	8 (5.7)	NA	NA	2 (1.6)	NA	NA	10 (3.8)	NA
Reclassified if threshold for sex assigned at birth was used^c^	NA	1 (0.7)	NA	NA	2 (1.6)	NA	NA	3 (1.1)	NA
Cause of abnormal troponin level									
Type 1 STEMI	1 (0.2)	1 (0.7)	.32	0	0	NA	1 (0.1)	1 (0.4)	.38
Type 1 NSTEMI	5 (0.9)	3 (2.1)	.26	1 (0.3)	0	.54	6 (0.7)	3 (1.1)	.49
Type 2 MI or nonischemic myocardial injury^d^	14 (2.6)	4 (2.9)	.91	3 (0.9)	4 (3.3)	.07	17 (2.0)	8 (3.1)	.32
30-d Clinical outcomes									
Death	3 (0.6)	0	.37	2 (0.6)	0	.39	5 (0.6)	0	.21
MI	8 (1.5)	4 (2.9)	.30	1 (0.3)	0	.54	9 (1.1)	4 (1.5)	.54
Heart failure	5 (0.9)	2 (1.4)	.63	0	0	NA	5 (0.6)	2 (0.8)	.76

Abbreviations: hs-cTnI, high-sensitivity cardiac troponin I; MI, myocardial infarction; NA, not applicable; NSTEMI, non–ST-segment elevation myocardial infarction; STEMI, ST-segment elevation myocardial infarction.

^a^ *P* values are reported for comparisons between the conventional troponin group and the hs-cTnI group. χ^2^ test was used.

^b^ For the conventional troponin assay, a single universal threshold for all patients was used (99th percentile value: 0.04 ng/mL). For the hs-cTnI assay, the 99th percentile threshold was 12 ng/L (0.012 ng/mL) for cisgender females and 20 ng/L (0.020 ng/mL) for cisgender males (to convert troponin I values from ng/mL to μg/L, multiply by 1).

^c^ Shows the number of patients who would be reclassified if troponin thresholds corresponding to their sex assigned at birth instead of their affirmed gender identity was used. Patients who were reclassified had elevated troponin levels due to cardiomyopathy with heart failure, sepsis, and supraventricular tachycardia.

^d^ Patients with type 2 MI or myocardial injury had elevated troponin levels due to sepsis, respiratory failure, gastrointestinal bleeding, heart failure, or tachyarrhythmia.

Three patients (1.1%), who would be reclassified if their sex assigned at birth were used for the threshold, had type 2 MI or myocardial injury. No patients with type 1 MI would have been reclassified. No significant differences in clinical outcomes at 30 days were observed before and after implementation of sex-specific troponin diagnostic thresholds.

## Discussion

In this cohort study of transgender patients, implementing sex-specific troponin thresholds for diagnosing AMI did not significantly affect outcomes. Use of sex-specific thresholds aligned with affirmed gender identity was not associated with significant reclassification. None of the few reclassified patients had myocardial injury due to type 1 MI. Patients presenting with ST-segment elevation MI and type 1 non–ST-segment elevation MI had troponin levels that were much higher than the diagnostic thresholds and were appropriately identified regardless of the threshold used.

Study limitations were short follow-up time, small sample size, and the observational design. The findings suggest that troponin thresholds in accordance with the patient’s affirmed gender identity may be used in transgender individuals. Doing so may reduce confusion and distress for both patients and clinicians and help patients receive truly gender-affirming care.

## Notes

Supplement.
Data Sharing Statement
