# Supplementary material for: Sex-Specific Cardiac Troponin Thresholds in Transgender Patients With Suspected Acute Coronary Syndrome
Source: JAMA Netw Open. 2023 Oct 12;6(10):e2337345. doi: 10.1001/jamanetworkopen.2023.37345 (PMC10570872; doi:10.1001/jamanetworkopen.2023.37345)
Supplement: Supplement. — Data Sharing Statement [file jamanetwopen-e2337345-s001.pdf]

Data Sharing Statement

Wang. Sex-Specific Cardiac Troponin Thresholds in Transgender Patients With Suspected Acute Coronary Syndrome. *JAMA Netw Open*. Published October 12, 2023. doi:10.1001/jamanetworkopen.2023.37345

Data
Data available: No
